# Prior-Guided Heterogeneous Dynamic Graph Convolutional Network for Bearing Fault Diagnosis

**DOI:** 10.3390/s26216678

**Published:** 2026-10-22

**Authors:** Zeyu Yang, Xin Wang, Wenqi Shao, Xiaolian Yang

**Affiliations:** 1 School of Robotics and Intelligent Manufacturing, Huzhou Normal University, Huzhou 313000, China; yangzeyu@zju.edu.cn (Z.Y.); 2025388645@stu.huznu.edu.cn (X.W.); 2026388623@stu.huznu.edu.cn (W.S.); 2 Huzhou Academy of Agricultural Sciences, Huzhou 313000, China

**Keywords:** bearing fault diagnosis, heterogeneous dynamic graph, graph convolutional network, prior-guided learning, frequency-domain representation

## Abstract

Reliable bearing fault diagnosis is important for the stable operation of rotating machinery under complex and varying conditions. Existing graph-based diagnostic methods commonly rely on either predefined fixed topologies or fully data-driven graph learning, making it difficult to balance structural reliability with state-dependent adaptability. To address this issue, this paper proposes a Prior-Guided Heterogeneous Dynamic Graph Convolutional Network (PHDGCN). PHDGCN constructs two relation-specific prior graphs from causality-consistent temporal dependencies and frequency-domain covariate correlations and uses them as structural anchors. A history-conditioned residual learning mechanism then refines the two priors according to the current local signal state while preserving their structural constraints. The refined temporal and covariate graphs are processed by independent graph convolutional branches, and their complementary representations are fused for fault classification. Experiments on two public bearing fault diagnosis datasets show that PHDGCN achieves accuracy/macro-F1 values of 93.75%/93.34% and 89.47%/87.08%, respectively, outperforming the compared conventional and graph-based methods. Ablation and graph-structure visualizations further show the contribution of prior-guided dynamic refinement and the relation-specific adjustment behavior of the two graph branches.

## 1. Introduction

With the development of industrial automation and intelligent manufacturing, rotating machinery is widely used in industrial systems, with bearings serving as critical components in many transmission and support mechanisms [1,2,3]. Load fluctuations, mechanical impacts, measurement noise, and environmental disturbances impose demanding reliability requirements on bearing systems [4,5]. Bearing degradation or failure can reduce operational stability, production efficiency, and equipment safety. Accurate bearing condition monitoring and fault diagnosis are therefore important for reducing unexpected downtime and improving equipment reliability [6,7].

Data-driven intelligent diagnosis has attracted increasing attention with the development of machine learning and deep learning [8]. Traditional approaches generally rely on handcrafted time-domain, frequency-domain, or time-frequency features followed by conventional classifiers, whereas deep models can learn discriminative representations directly from vibration signals [9,10,11,12]. However, most conventional deep architectures operate on regular Euclidean structures and do not explicitly model irregular dependencies among temporal states and signal variables [13,14].

Graph neural networks provide a flexible framework for representing such dependencies through nodes, edges, and message passing [15,16]. In bearing fault diagnosis and industrial process modeling, graph construction can generally be viewed from three perspectives. Similarity-based graphs establish connections according to distances, similarities, or statistical correlations [17,18]. Temporal graphs encode dependencies according to sampling order or historical relationships [19,20]. Covariate graphs represent sensor variables or signal channels as nodes, with edges encoding inter-variable dependencies based on statistical correlations or known physical relationships [12,21]. However, a single relation usually characterizes only one aspect of the underlying dependency mechanism. Temporal graphs describe state evolution but do not directly model interactions among variables, whereas covariate graphs describe inter-variable relationships but may not capture their temporal variation. Multi-relational or heterogeneous graph representations can therefore provide complementary structural views [22,23]. Nevertheless, many existing graph-based diagnostic methods rely on predefined or offline-estimated topologies that remain fixed during inference.

Dynamic graph learning improves topology adaptability by allowing the adjacency matrix to depend on the observed state [24,25,26,27]. Existing approaches commonly infer graph connections through learnable embeddings, attention mechanisms, similarity functions, or neural networks [20,23,28,29]. Fully data-driven topology learning, however, may become unstable when the available data are limited or noisy, and unconstrained topology estimation can introduce relationships that lack clear temporal or statistical meaning [30].

In many vibration-monitoring systems, useful relational information is already available from temporal continuity and statistical dependencies. Such information may not provide the optimal topology for every local signal state, but it can serve as a reliable structural reference [31]. This motivates a prior-guided strategy in which predefined relationships are treated as structural anchors and only state-dependent residual adjustments are learned. In this way, graph adaptation is restricted to a prior-centered neighborhood rather than being learned freely from scratch.

Based on this idea, this paper proposes a **Prior-Guided Heterogeneous Dynamic Graph Convolutional Network (PHDGCN)** for bearing fault diagnosis. Vibration signals are first converted into local frequency-domain representations. A causal temporal prior and a correlation-based covariate prior are then constructed to describe complementary dependencies. The current and recent frequency-domain states are jointly used to estimate relation-specific topology residuals, which refine the two prior graphs. Finally, separate graph convolutional branches extract temporal and covariate representations, which are fused for fault classification.

The main contributions are summarized as follows:

1. A heterogeneous prior graph framework is developed to jointly model temporal dependencies and inter-variable correlations in bearing vibration signals.
2. A prior-guided dynamic refinement mechanism is proposed to adapt the temporal and covariate graphs to local signal states while preserving their structural constraints.

The remainder of this paper is organized as follows. Section 2 introduces the problem formulation and graph representation. Section 3 presents the proposed PHDGCN methodology. Section 4 reports the datasets, experimental settings, comparison results, ablation study, parameter sensitivity analysis, graph-refinement visualization, and internal processing visualization. Section 5 concludes the paper.

## 2. Preliminaries

### 2.1. Problem Formulation

Let a multivariate vibration recording be denoted by $X={\left[x_{1},x_{2},\ldots ,x_{N}\right]}^{⊤}\in R^{N\times D}$, where *N* is the number of sampling points, *D* is the number of synchronized variables or signal channels, and $x_{n}\in R^{D}$ is the observation at the *n*th sampling point. After segmentation and frequency-domain transformation, the diagnostic input at index *t* is represented by a current–historical tensor $S_{t}$. The objective is to learn a classifier $g_{\Theta }$ that maps $S_{t}$ to the class-probability vector $p_{t}\in R^{C}$:

(1) $$p_{t}=g_{\Theta }\left(S_{t}\right),\;{\hat{y}}_{t}=\arg\max_{c}p_{t,c},$$

where *C* is the number of diagnostic classes and ${\hat{y}}_{t}$ is the predicted class label.

### 2.2. Heterogeneous Graph Representation

A relation-specific graph is represented as

(2) $$G^{r}=\left(V^{r},E^{r},A^{r},X^{r}\right),\;r\in R,$$

where $V^{r}$ and $E^{r}$ denote the node and edge sets, respectively; $A^{r}\in R^{n_{r}\times n_{r}}$ denotes the adjacency matrix; and $X^{r}\in R^{n_{r}\times d_{r}}$ denotes the node feature matrix. In this work, R={tem,cov}. The temporal graph models dependencies among historical frequency-domain states, whereas the covariate graph models dependencies among variables within the current frequency-domain state [23].

### 2.3. Graph Convolution

For either relation *r*, PHDGCN adopts a message-passing operator that separately transforms the node itself and the aggregated neighbor message. Given the normalized adjacency matrix ${\hat{A}}_{t}^{r}$ and node representation $H_{t}^{r,(l)}$, the *l*th graph convolutional layer is

(3) $$H_{t}^{r,(l+1)}=\sigma \left[\mathrm{LN}\left(H_{t}^{r,(l)}W_{s}^{r,(l)}+{\hat{A}}_{t}^{r}H_{t}^{r,(l)}W_{m}^{r,(l)}\right)\right],$$

where $W_{s}^{r,(l)}$ and $W_{m}^{r,(l)}$ are trainable self-feature and message-transformation matrices, LN(·) denotes layer normalization, and σ(·) is the ReLU activation function [16].

## 3. Methodology

### 3.1. Overall Framework

**Figure 1:**
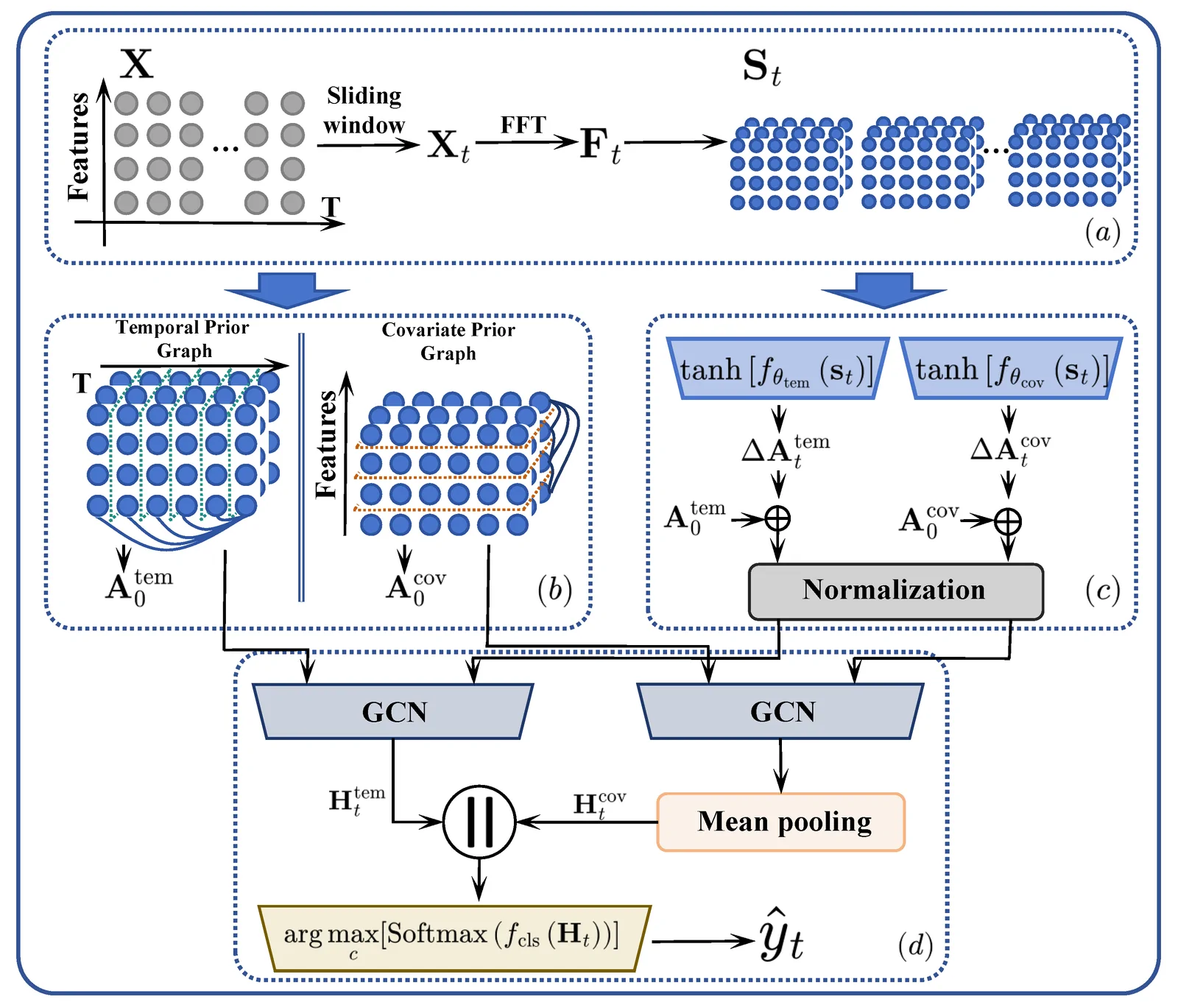
Overall framework of the proposed PHDGCN: (**a**) signal segmentation and frequency-domain representation; (**b**) heterogeneous prior graph construction; (**c**) dynamic graph refinement; (**d**) heterogeneous graph convolution and fusion prediction.

PHDGCN consists of four stages: signal segmentation and frequency-domain representation, heterogeneous prior graph construction, dynamic graph refinement, and heterogeneous graph convolution with feature fusion. The continuous vibration signal is first divided into local windows, and each variable is transformed into a one-sided FFT magnitude spectrum. The current frequency-domain window and its preceding *K* windows form a current–historical representation. Temporal and covariate prior graphs are then constructed to describe causality-consistent temporal dependencies and inter-variable statistical dependencies, respectively. Two relation-specific residual networks refine the prior graphs according to the current local signal state. Finally, the refined graphs are processed by independent graph convolutional branches, and their representations are fused for fault classification. The overall framework is illustrated in Figure 1.

### 3.2. Signal Segmentation and Frequency-Domain Representation

Given a multivariate vibration signal $X={\left[x_{1},x_{2},\ldots ,x_{N}\right]}^{⊤}\in R^{N\times D}$, where *D* denotes the number of signal variables, the *t*th local window with length *L* and stride *s* is

(4) $$X_{t}={\left[x_{(t-1)s+1},x_{(t-1)s+2},\ldots ,x_{(t-1)s+L}\right]}^{⊤}\in R^{L\times D}.$$

Before FFT extraction, each raw variable is standardized using statistics estimated from the training partition only. For the *d*th variable in the *t*th window, the one-sided discrete Fourier transform is

(5) $${\tilde{F}}_{t}^{\left(d\right)}\left(k\right)=\sum_{n=0}^{L-1}x_{t}^{\left(d\right)}\left(n\right)\exp\left(-j\frac{2\pi kn}{L}\right),$$

and the corresponding magnitude is

(6) $$F_{t}^{\left(d\right)}\left(k\right)=\frac{1}{L}\left|{\tilde{F}}_{t}^{\left(d\right)}\left(k\right)\right|.$$

A logarithmic transformation is subsequently applied to compress the dynamic range of the spectral amplitudes:

(7) $${\bar{F}}_{t}^{\left(d\right)}\left(k\right)=\log\left(1+F_{t}^{\left(d\right)}\left(k\right)\right).$$

After standardization using training-set statistics, the resulting frequency-domain representation of the *t*th window is denoted by

(8) $$F_{t}\in R^{D\times F},\;F=\left\lfloor \frac{L}{2}\right\rfloor +1,$$

where *F* is the number of retained frequency bins.

To incorporate recent temporal information, the current window and its preceding *K* frequency-domain windows are organized as

(9) $$S_{t}=\left[F_{t-K},F_{t-K+1},\ldots ,F_{t}\right]\in R^{(K+1)\times D\times F}.$$

### 3.3. Heterogeneous Prior Graph Construction

PHDGCN constructs two relation-specific prior graphs:

(10) $$G_{0}^{r}=\left(V^{r},E_{0}^{r},A_{0}^{r}\right),\;r\in \left\{\mathrm{tem},\mathrm{cov}\right\}.$$

The node features of both graphs are derived from the frequency-domain representations $F_{t}$. The temporal graph describes causal dependencies among consecutive frequency-domain states, whereas the covariate graph characterizes statistical dependencies among different signal variables.

#### 3.3.1. Temporal Prior Graph

For sample *t*, the temporal node set is defined as

(11) $$V_{t}^{\mathrm{tem}}=\left\{v_{t-K}^{\mathrm{tem}},v_{t-K+1}^{\mathrm{tem}},\ldots ,v_{t}^{\mathrm{tem}}\right\},$$

where each node $v_{q}^{\mathrm{tem}}$ corresponds to the frequency-domain representation $F_{q}$, with q∈{t−K,…,t}.

Let *i* and *j* denote the local target and source node indices, respectively. To preserve temporal causality, the temporal prior adjacency matrix is defined as

(12) $${\left[A_{0}^{\mathrm{tem}}\right]}_{ij}=\left\{\begin{array}{cc} \exp\left(-\frac{i-j}{\tau }\right),\; & j<i,\\ 0,\; & j\geq i, \end{array}\right.$$

where τ>0 controls the decay of dependency strength with temporal distance.

For samples with incomplete historical information, a validity mask is introduced:

(13) $$M_{t,ij}^{\mathrm{tem}}=m_{t,i}m_{t,j}I\left(j<i\right),$$

where $m_{t,i}\in \left\{0,1\right\}$ indicates whether the *i*th temporal node is valid. This constraint prevents padded nodes and future information from participating in temporal message passing.

#### 3.3.2. Covariate Prior Graph

The covariate node set is defined as

(14) $$V^{\mathrm{cov}}=\left\{v_{1}^{\mathrm{cov}},v_{2}^{\mathrm{cov}},\ldots ,v_{D}^{\mathrm{cov}}\right\}.$$

Each node corresponds to one signal variable, and the *i*th node uses $F_{t}^{\left(i\right)}\in R^{F}$ as its frequency-domain feature.

To construct a stable covariate prior, the training FFT features of variable *i* are concatenated across training windows and frequency bins into $z_{i}=\left[z_{i,1},z_{i,2},\ldots ,z_{i,M}\right]$. The Pearson correlation coefficient between variables *i* and *j* is calculated as

(15) $$\rho_{ij}=\frac{\sum_{n=1}^{M}\left(z_{i,n}-{\bar{z}}_{i}\right)\left(z_{j,n}-{\bar{z}}_{j}\right)}{\sqrt{\sum_{n=1}^{M}{\left(z_{i,n}-{\bar{z}}_{i}\right)}^{2}}\sqrt{\sum_{n=1}^{M}{\left(z_{j,n}-{\bar{z}}_{j}\right)}^{2}}}.$$

Here, $\rho_{ij}\in \left[-1,1\right]$ denotes the Pearson correlation coefficient between variables *i* and *j*, whose magnitude reflects the strength of their linear correlation [32].

The covariate prior adjacency matrix is then defined as

(16) $${\left[A_{0}^{\mathrm{cov}}\right]}_{ij}=\left\{\begin{array}{cc} \frac{\rho_{ij}}{\max_{p\neq q}\left|\rho_{pq}\right|},\; & i\neq j,\\ 0,\; & i=j. \end{array}\right.$$

This formulation retains both positive and negative statistical dependencies while excluding self-connections.

### 3.4. Dynamic Graph Refinement

The current–historical frequency-domain representation is first vectorized as

(17) $$s_{t}=\mathrm{vec}\left(S_{t}\right)\in R^{(K+1)DF}.$$

Two independent two-layer MLPs estimate the temporal and covariate topology residuals:

(18) $$\Delta A_{t}^{\mathrm{tem}}=\tanh\left[f_{\theta_{\mathrm{tem}}}\left(s_{t}\right)\right],\;\Delta A_{t}^{\mathrm{cov}}=\tanh\left[f_{\theta_{\mathrm{cov}}}\left(s_{t}\right)\right].$$

The final linear layer of each residual network is zero-initialized so that graph refinement starts from the corresponding prior structure.

For the temporal graph, the residual is restricted to valid causal edges:

(19) $$\Delta {\bar{A}}_{t}^{\mathrm{tem}}=\Delta A_{t}^{\mathrm{tem}}⊙M_{t}^{\mathrm{tem}},$$

and the refined temporal adjacency matrix is

(20) $$A_{t}^{\mathrm{tem}}=A_{0}^{\mathrm{tem}}⊙\left(1+\lambda_{\mathrm{tem}}\Delta {\bar{A}}_{t}^{\mathrm{tem}}\right)⊙M_{t}^{\mathrm{tem}},$$

where $\lambda_{\mathrm{tem}}$ controls the temporal refinement strength.

For the covariate graph, the predicted residual is first symmetrized:

(21) $$\Delta {\bar{A}}_{t}^{\mathrm{cov}}=\frac{1}{2}\left[\Delta A_{t}^{\mathrm{cov}}+{\left(\Delta A_{t}^{\mathrm{cov}}\right)}^{⊤}\right],\;\mathrm{diag}\left(\Delta {\bar{A}}_{t}^{\mathrm{cov}}\right)=0.$$

The refined covariate adjacency matrix is then obtained as

(22) $$A_{t}^{\mathrm{cov}}=A_{0}^{\mathrm{cov}}+\lambda_{\mathrm{cov}}\left(\left|A_{0}^{\mathrm{cov}}\right|+\epsilon_{c}\right)⊙\Delta {\bar{A}}_{t}^{\mathrm{cov}},$$

where $\lambda_{\mathrm{cov}}$ controls the covariate refinement strength and $\epsilon_{c}$ allows limited adjustment of weak prior connections.

Before graph convolution, each refined adjacency matrix is normalized according to its absolute row sum:

(23) $$d_{t,i}^{r}=\max\left(\sum_{q}\left|A_{t,iq}^{r}\right|,\epsilon_{n}\right),\;{\hat{A}}_{t,ij}^{r}=\frac{A_{t,ij}^{r}}{d_{t,i}^{r}},$$

where r∈{tem,cov} and $\epsilon_{n}$ is a small constant for numerical stability.

### 3.5. Heterogeneous Graph Convolution and Fusion Prediction

After dynamic refinement, the temporal and covariate graphs are processed by two independent graph convolutional branches using ${\hat{A}}_{t}^{\mathrm{tem}}$ and ${\hat{A}}_{t}^{\mathrm{cov}}$, respectively.

For the temporal branch, each frequency-domain window $F_{q}\in R^{D\times F}$, where q∈{t−K,…,t}, is flattened and projected into an *H*-dimensional graph feature space. The initial temporal node feature matrix is represented as

(24) $$H_{t}^{\mathrm{tem},(0)}={\left[h_{t-K}^{\mathrm{tem},(0)},\ldots ,h_{t}^{\mathrm{tem},(0)}\right]}^{⊤}\in R^{(K+1)\times H}.$$

Using the refined temporal adjacency matrix, the two graph convolutional layers are expressed as

(25) $$H_{t}^{\mathrm{tem},(l+1)}=\sigma \left[\mathrm{LN}\left(H_{t}^{\mathrm{tem},(l)}W_{s}^{\mathrm{tem},(l)}+{\hat{A}}_{t}^{\mathrm{tem}}H_{t}^{\mathrm{tem},(l)}W_{m}^{\mathrm{tem},(l)}\right)\right],\;l=0,1.$$

After graph propagation, the representation of the current temporal node is selected as the temporal branch output:

(26) $$H_{t}^{\mathrm{tem}}=h_{t}^{\mathrm{tem},(2)}\in R^{H}.$$

For the covariate branch, each node corresponds to one signal variable and directly uses its *F*-dimensional frequency-domain feature $F_{t}^{\left(i\right)}$. The features are projected into the *H*-dimensional graph feature space, with a learnable variable-identity embedding incorporated to distinguish different signal variables. The initial covariate node feature matrix is

(27) $$H_{t}^{\mathrm{cov},(0)}={\left[h_{t,1}^{\mathrm{cov},(0)},\ldots ,h_{t,D}^{\mathrm{cov},(0)}\right]}^{⊤}\in R^{D\times H}.$$

Using the refined covariate adjacency matrix, the two graph convolutional layers are expressed as

(28) $$H_{t}^{\mathrm{cov},(l+1)}=\sigma \left[\mathrm{LN}\left(H_{t}^{\mathrm{cov},(l)}W_{s}^{\mathrm{cov},(l)}+{\hat{A}}_{t}^{\mathrm{cov}}H_{t}^{\mathrm{cov},(l)}W_{m}^{\mathrm{cov},(l)}\right)\right],\;l=0,1.$$

After two graph convolutional layers, the final covariate node representations are aggregated by mean pooling:

(29) $$H_{t}^{\mathrm{cov}}=\frac{1}{D}\sum_{i=1}^{D}h_{t,i}^{\mathrm{cov},(2)}\in R^{H}.$$

The temporal and covariate representations are then concatenated as

(30) $$H_{t}=H_{t}^{\mathrm{tem}}∥H_{t}^{\mathrm{cov}}\in R^{2H}.$$

The fused representation is fed into the classification head, and the class probabilities and predicted fault label are obtained as

(31) $$p_{t}=\mathrm{Softmax}\left(f_{\mathrm{cls}}\left(H_{t}\right)\right),\;{\hat{y}}_{t}=\arg\max_{c}p_{t,c}.$$

where $f_{\mathrm{cls}}\left(\cdot \right)$ denotes a two-layer MLP classification head; $p_{t}$ denotes the predicted class-probability vector; and ${\hat{y}}_{t}$ denotes the predicted fault label.

### 3.6. Training Objective

The proposed model is optimized using a weighted cross-entropy loss together with topology residual regularization:

(32) $$L_{\mathrm{cls}}=-\sum_{c=1}^{C}w_{c}y_{c}\log p_{c},$$

where *C* denotes the number of classes; $w_{c}$ is the class weight; and $y_{c}$ and $p_{c}$ denote the ground-truth indicator and predicted probability of class *c*, respectively.

To constrain excessive deviations from the prior graphs, the temporal and covariate topology residuals are regularized as

(33) $$L_{\mathrm{tem}}^{\mathrm{reg}}=\frac{{∥\Delta A^{\mathrm{tem}}⊙M^{\mathrm{tem}}∥}_{F}^{2}}{{∥M^{\mathrm{tem}}∥}_{1}},\;L_{\mathrm{cov}}^{\mathrm{reg}}=\frac{{∥\Delta {\bar{A}}^{\mathrm{cov}}∥}_{F}^{2}}{D(D-1)}.$$

The overall objective is

(34) $$L=L_{\mathrm{cls}}+\beta \left(L_{\mathrm{tem}}^{\mathrm{reg}}+L_{\mathrm{cov}}^{\mathrm{reg}}\right),$$

where β controls the strength of topology residual regularization.

## 4. Case Studies

### 4.1. Dataset Description

Two publicly available bearing fault datasets are used to evaluate PHDGCN: the Case Western Reserve University (CWRU) bearing dataset [33] and the Korea Advanced Institute of Science and Technology (KAIST) rotating machinery fault dataset [34]. The selected subsets contain multiple fault types and severity levels and are used as two complementary diagnostic benchmarks.

#### 4.1.1. CWRU Dataset

For CWRU, the 12-kHz drive-end bearing signals under the no-load condition are selected. The subset contains normal samples, ball faults, inner-race faults, and outer-race faults with different defect sizes and, for outer-race faults, different fault positions. Each listed operating condition is treated as an independent diagnostic class, resulting in 14 classes as summarized in Table 1.

The CWRU experiment uses two synchronized vibration signals measured at the drive end and fan end as inputs (D=2), as summarized in Table 2.

The two variables are standardized independently using the mean and standard deviation estimated from the training partition only, and the same statistics are applied to the validation and test partitions.

#### 4.1.2. KAIST Dataset

For KAIST, vibration signals collected under the 0-Nm load condition are used. The selected subset contains bearing inner-race faults, bearing outer-race faults, shaft misalignment, rotor unbalance, and normal condition. Different fault severity levels are treated as independent classes, resulting in 9 diagnostic classes as summarized in Table 3.

The KAIST experiment uses four synchronized vibration channels measured along the *x*- and *y*-directions of bearing housings A and B as inputs (D=4), as summarized in Table 4.

The four variables are standardized independently using training-set statistics, which are then applied unchanged to the validation and test partitions.

### 4.2. Dataset Construction and Data Splitting

The original recordings are organized by operating or fault condition. To obtain multiple continuous local sequences while preserving the original temporal order, class-wise recordings are sampled into random contiguous segments. Let the signal of class *c* be

(35) $$X^{\left(c\right)}={\left[x_{1}^{\left(c\right)},x_{2}^{\left(c\right)},\ldots ,x_{N_{c}}^{\left(c\right)}\right]}^{⊤}.\;$$

For the *m*th sampled segment, a class $c_{m}$ is first selected, and its segment length $\ell_{m}$ is randomly sampled from the discrete interval [128,256], all segments are sampled without replacement, ensuring that no raw samples are reused across different segments:

(36) $$\ell_{m}∼U\left\{128,129,\ldots ,256\right\}.$$

Given the selected class and segment length, a valid starting position $s_{m}$ is randomly sampled as

(37) $$s_{m}∼U\left\{1,2,\ldots ,N_{c_{m}}-\ell_{m}+1\right\}.$$

The corresponding contiguous segment is then extracted as

(38) $$Q_{m}={\left[x_{s_{m}}^{\left(c_{m}\right)},x_{s_{m}+1}^{\left(c_{m}\right)},\ldots ,x_{s_{m}+\ell_{m}-1}^{\left(c_{m}\right)}\right]}^{⊤}.$$

No permutation is applied within each segment, so the original local temporal order is preserved.

Because the original recordings are collected separately under different fault conditions rather than along a single continuous operating timeline, the randomly extracted segments are used to construct a surrogate operating sequence with varying fault occurrences. For each sampled segment, the original internal temporal order is strictly preserved, while its starting position and length are randomly determined to introduce variability in the occurrence position and duration of different fault conditions. The sampled segments are then concatenated according to their extraction order to form the final diagnostic sequence. Finally, the resulting sequence is divided chronologically into training, validation, and test sets with a ratio of 60%:20%:20% (3:1:1), without random shuffling, so that the temporal order of the constructed sequence is preserved throughout model development and evaluation.

### 4.3. Parameter Settings

The same PHDGCN architecture is adopted for both datasets, while several data-processing and training parameters are adjusted according to the characteristics of CWRU and KAIST. The model is trained using the Adam optimizer, and early stopping is performed according to the validation Macro-F1 score. The complete parameter configuration is summarized in Table 5.

### 4.4. Evaluation Metrics

Classification performance is evaluated using accuracy (ACC) and macro-averaged F1-score (F1). For $N_{\mathrm{te}}$ test samples, ACC is defined as

(39) $$\mathrm{ACC}=\frac{1}{N_{\mathrm{te}}}\sum_{i=1}^{N_{\mathrm{te}}}I\left({\hat{y}}_{i}=y_{i}\right),$$

where ${\hat{y}}_{i}$ and $y_{i}$ denote the predicted and ground-truth labels of the *i*th sample, respectively, and I(·) denotes the indicator function.

The macro-averaged F1-score is defined as

(40) $$F1=\frac{1}{C}\sum_{c=1}^{C}\frac{2{\mathrm{TP}}_{c}}{2{\mathrm{TP}}_{c}+{\mathrm{FP}}_{c}+{\mathrm{FN}}_{c}},$$

where *C* denotes the number of fault classes, and ${\mathrm{TP}}_{c}$, ${\mathrm{FP}}_{c}$, and ${\mathrm{FN}}_{c}$ denote the true-positive, false-positive, and false-negative counts for class *c*, respectively. Higher ACC and F1 values indicate better classification performance.

### 4.5. Comparison Experiments

PHDGCN is compared with several representative methods, including MLP, LSTM, CNN, GCN [35], TCN [36], SENGraph [28], and ASG-GTCN [29]. The key parameter settings of the comparison methods are summarized in Table 6. The comparison results on the CWRU and KAIST datasets are summarized in Table 7.

All comparison models are trained using the Adam optimizer with a learning rate of $10^{-3}$, a weight decay of $10^{-5}$, a maximum of 200 epochs, and an early-stopping patience of 35.

As shown in Table 7, PHDGCN achieves the highest ACC and F1 on both datasets. On CWRU, PHDGCN improves ACC and F1 by 3.11 and 3.37 percentage points, respectively, compared with the strongest conventional baseline. On KAIST, the corresponding improvements are 4.81 and 2.74 percentage points. Compared with ASG-GTCN, PHDGCN improves ACC/F1 by 4.52/4.68 percentage points on CWRU and 5.11/4.67 percentage points on KAIST. In the considered deployment setting, model training is performed offline, while only the trained model is deployed on the edge device for inference. PHDGCN requires 3.12 ms and 2.94 ms per sample on CWRU and KAIST, respectively, and remains faster than ASG-GTCN on both datasets while maintaining millisecond-level latency. These results demonstrate the effectiveness of combining structural priors with state-dependent graph refinement for bearing fault diagnosis.

### 4.6. Ablation Study

Ablation experiments evaluate the contribution of the dynamic refinement mechanisms and the two relation-specific branches. A1 removes temporal dynamic refinement; A2 removes covariate dynamic refinement; A3 retains both relations but fixes their graph structures; and A4 and A5 retain only the temporal or covariate branch, respectively. The results are summarized in Table 8.

Among the ablation variants, A3 provides the most direct assessment of the proposed dynamic refinement mechanism because it preserves the same dual-view prior structure and graph branches while disabling state-dependent topology refinement. Relative to A3, the full model improves ACC/F1 by 5.45/6.03 percentage points on CWRU and by 8.18/8.42 percentage points on KAIST, supporting the effectiveness of prior-guided dynamic refinement. In addition, removing temporal refinement (A1) or covariate refinement (A2) independently also leads to clear degradation, indicating that both relation-specific dynamic adjustments contribute to the final prediction. The single-branch variants A4 and A5 further demonstrate the complementary roles of the temporal and covariate representations.

### 4.7. Parameter Sensitivity Analysis

Parameter sensitivity is evaluated on CWRU by varying one parameter at a time while fixing the remaining parameters at their default values. The investigated parameters are the history length *K*, temporal decay coefficient τ, temporal refinement coefficient $\lambda_{\mathrm{tem}}$, covariate refinement coefficient $\lambda_{\mathrm{cov}}$, topology regularization coefficient β, graph hidden dimension *H*, and FFT window length *L*. Test ACC is reported only for sensitivity analysis; the dashed line in each panel marks the default configuration rather than the test-set optimum.

**Figure 2:**
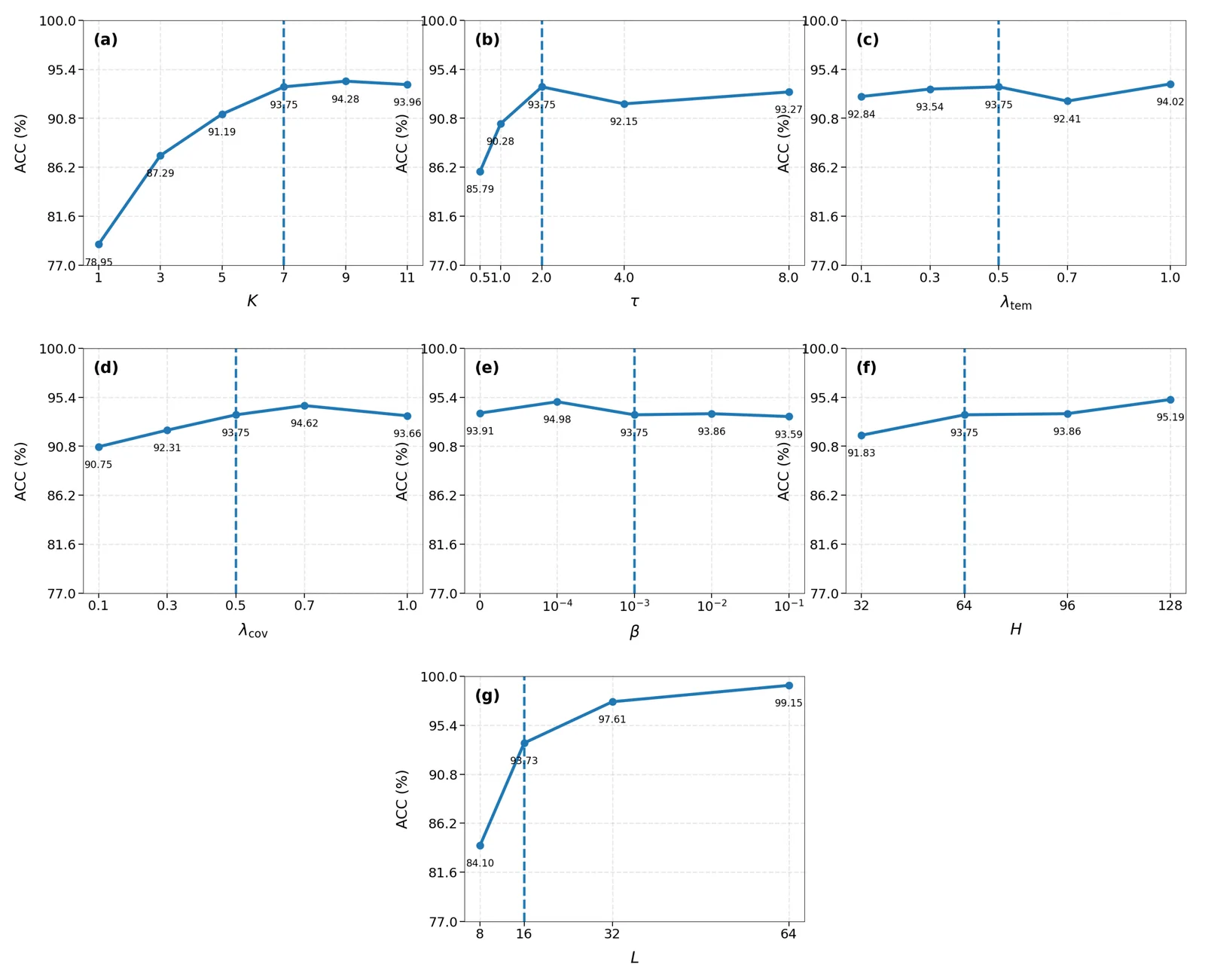
Parameter sensitivity analysis of PHDGCN on the CWRU dataset: (**a**) historical window number *K*; (**b**) temporal decay coefficient τ; (**c**) temporal refinement coefficient $\lambda_{\mathrm{tem}}$; (**d**) covariate refinement coefficient $\lambda_{\mathrm{cov}}$; (**e**) topology regularization coefficient β; (**f**) graph hidden dimension *H*; (**g**) FFT window length *L*. The dashed lines indicate the default parameter settings specified in Table 5.

Figure 2 shows that the history length has a pronounced influence on performance. ACC increases from 78.95% at K=1 to 94.28% at K=9, followed by a slight decrease at K=11, indicating that sufficient but not excessive temporal context is beneficial. The temporal decay coefficient produces its best reported ACC around τ=2, whereas the two refinement coefficients show non-monotonic behavior, confirming that overly weak or strong topology adjustment is not uniformly beneficial.

The model is comparatively less sensitive to β within the evaluated range, with ACC remaining between 93.59% and 94.98%. Increasing *H* from 32 to 128 improves ACC from 91.83% to 95.19%, indicating that representation capacity has a noticeable effect. For the FFT window length, ACC increases from 84.10% at L=8 to 99.15% at L=64, indicating that longer windows provide richer frequency-domain information. Nevertheless, L=16 is retained as the default setting in the main experiments as a compact configuration rather than the performance-optimal choice. Overall, the sensitivity results show that PHDGCN remains usable over a broad parameter range, while the history length and FFT window length have the most visible influence in the reported experiments.

### 4.8. Internal Processing Visualization

**Figure 3:**
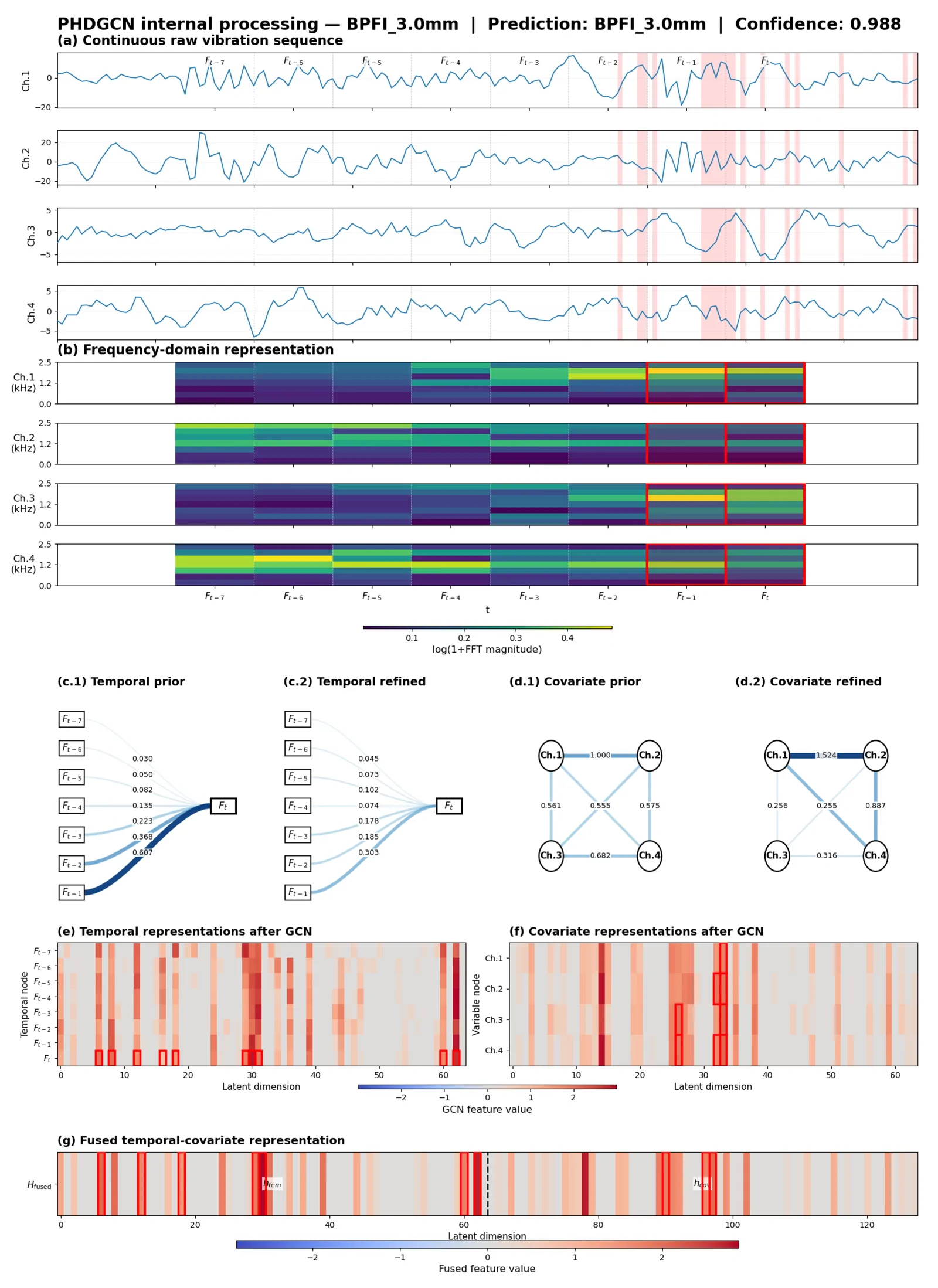
Internal processing visualization for a representative BPFI 3.0 mm sample. (**a**) Original multichannel vibration signals; (**b**) frequency-domain historical representations; (**c.1**,**c.2**) temporal prior and refined graphs; (**d.1**,**d.2**) covariate prior and refined graphs; (**e**,**f**) temporal and covariate representations after graph convolution; (**g**) fused representation. The red regions and boxes highlight components with relatively high contribution to the predicted class.

**Figure 4:**
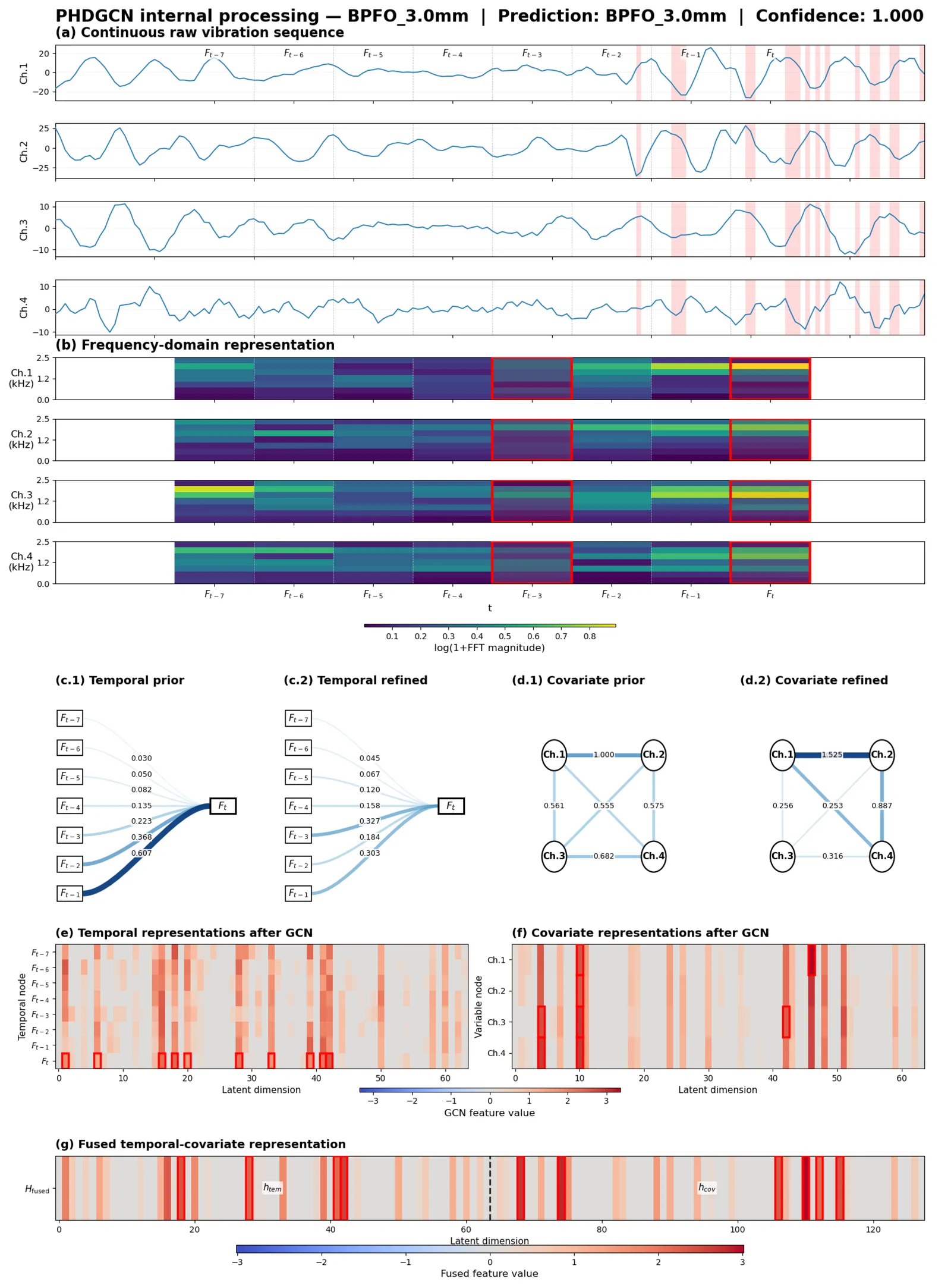
Internal processing visualization for a representative BPFO 3.0 mm sample. The subfigures are described as follows: (**a**) Original multichannel vibration signals; (**b**) frequency-domain historical representations; (**c.1**,**c.2**) temporal prior and refined graphs; (**d.1**,**d.2**) covariate prior and refined graphs; (**e**,**f**) temporal and covariate representations after graph convolution; (**g**) fused representation. The red regions and boxes highlight components with relatively high contribution to the predicted class.

Figure 3 and Figure 4 show two representative KAIST examples, using BPFI 3.0 mm and BPFO 3.0 mm samples to illustrate the internal processing of PHDGCN. From (a) to (b), the multichannel vibration signals are transformed into frequency-domain representations according to Equations (5)–(7), and consecutive windows are organized into the historical representation $S_{t}$ according to Equation (9). From (b) to (c)–(d), the temporal and covariate priors are adjusted by the state-dependent refinement mechanisms in Equations (20)–(22). The refined graphs are then processed by graph convolution to obtain the representations in (e) and (f), which are concatenated according to Equation (30) to form the fused representation in (g).

To better reveal how dynamic graph refinement affects the prediction, attribution is calculated with respect to the predicted-class probability $p_{t,c}$ [37]:

(41) $$I_{n}^{\mathrm{raw}}=\sum_{d=1}^{D}\left|\frac{\partial p_{t,c}}{\partial x_{n,d}}\right|,\;I_{q}^{\mathrm{FFT}}=\sum_{d=1}^{D}\sum_{k=1}^{F}\left|\frac{\partial p_{t,c}}{\partial S_{t,q,d,k}}\right|,\;I_{i}^{\mathrm{GA}}=\left|h_{i}\frac{\partial p_{t,c}}{\partial h_{i}}\right|.$$

The red regions and boxes highlight components with relatively high contribution to the predicted class. In PHDGCN, these differences are influenced by both the input features and the dynamically refined graph structures, which adjust the message-passing strengths among historical windows and signal channels. Therefore, the highlighted components indicate information that contributes more strongly to the current fault decision.

As shown in Figure 3, the refined temporal graph increases the weight of $F_{t-5}$ relative to the temporal prior, while $F_{t-1}$ and $F_{t-2}$ receive comparable weights. In Figure 4, the weight of $F_{t-3}$ increases from 0.223 to 0.327, becoming the strongest temporal connection, while the weight of $F_{t-1}$ decreases from 0.607 to 0.303. These differences suggest that the fixed temporal prior cannot fully capture the state-dependent temporal relationships of individual samples. Meanwhile, the refined graphs still retain the basic prior tendency that temporally closer nodes receive stronger connections, while allowing individual relationships to be adaptively adjusted according to the current vibration state.

The covariate graphs provide complementary information by describing the relationships among different signal channels. As shown in Figure 3 and Figure 4, the strong Ch.1–Ch.2 relationship in the covariate prior remains prominent after refinement, while the strengths of the other inter-channel connections are also retained with different relative magnitudes. This indicates that the diagnostic representation is not formed from each channel independently; instead, information from mutually related channels can be jointly propagated through the covariate graph. This is useful for bearing fault diagnosis because a fault-related vibration pattern may be reflected not only by the response of an individual channel, but also by the joint variation and relationship among multiple measured channels. By aggregating information along these cross-channel relationships, the covariate branch allows the model to consider the consistency and complementarity of different measurements, which can provide more discriminative information for distinguishing bearing states.

Therefore, the temporal and covariate graphs contribute to diagnosis from different but complementary perspectives. The temporal graph emphasizes how the vibration state evolves across historical observations, whereas the covariate graph emphasizes how different measured channels jointly characterize the current vibration state. The dynamic refinement combines these two forms of information by preserving the stable structural constraints of the priors while adapting the corresponding message-passing strengths to the current sample. The resulting representations can consequently incorporate both temporal evolution and cross-channel relationships, providing a more informative basis for the final fault classification.

## 5. Conclusions

This paper proposed PHDGCN, a prior-guided heterogeneous dynamic graph convolutional network for bearing fault diagnosis. The method represents temporal and covariate dependencies with two relation-specific prior graphs and refines them using bounded residuals estimated from current and historical FFT features. The refined graphs are processed by independent GCN branches and fused for classification, allowing the model to preserve meaningful structural constraints while adapting edge strengths to the local signal state.

On CWRU and KAIST, PHDGCN achieved ACC/F1 values of 93.75%/93.34% and 89.47%/87.08%, respectively, outperforming all compared methods in the reported experiments. Relative to the fixed dual-view variant, dynamic refinement improved ACC/F1 by 5.45/6.03 percentage points on CWRU and 8.18/8.42 percentage points on KAIST. The graph-structure visualizations further showed that temporal and covariate priors are retained as structural anchors while their edge strengths are adjusted in a relation-specific manner. The internal-processing visualization further shows that different fault samples exhibit distinct temporal-window and latent-feature contributions throughout the prediction process.

The current study is limited to the selected benchmark conditions. Future work will evaluate the method under broader load and noise conditions, larger cross-condition test sets, and cross-domain scenarios, and will further investigate the physical interpretation and stability of the learned graph refinements.

## Figures and Tables

**Table 1:** Selected diagnostic classes from the CWRU dataset.

Class	Fault Type	Fault Condition
1	Normal	Healthy condition
2	Ball fault	0.007 in
3	Ball fault	0.014 in
4	Ball fault	0.021 in
5	Inner-race fault	0.007 in
6	Inner-race fault	0.014 in
7	Inner-race fault	0.021 in
8	Outer-race fault	0.007 in, 3 o’clock
9	Outer-race fault	0.007 in, 6 o’clock
10	Outer-race fault	0.007 in, 12 o’clock
11	Outer-race fault	0.014 in, 6 o’clock
12	Outer-race fault	0.021 in, 3 o’clock
13	Outer-race fault	0.021 in, 6 o’clock
14	Outer-race fault	0.021 in, 12 o’clock

Note: “O’clock” denotes the angular position of the outer-race fault around the bearing circumference according to the conventional clock-face reference, with 12 o’clock corresponding to the top position.

**Table 2:** Input variables used for the CWRU dataset.

Input Variable	Signal Description	Sampling Frequency
$X_{1}$	Drive-end vibration signal	12 kHz
$X_{2}$	Fan-end vibration signal	12 kHz

**Table 3:** Selected diagnostic classes from the KAIST dataset.

Class	Fault Type	Fault Condition
1	Inner-race fault (BPFI)	0.3 mm
2	Inner-race fault (BPFI)	3.0 mm
3	Outer-race fault (BPFO)	0.3 mm
4	Outer-race fault (BPFO)	3.0 mm
5	Misalignment	0.1 mm
6	Misalignment	0.5 mm
7	Normal	Healthy condition
8	Unbalance	583 mg
9	Unbalance	3318 mg

**Table 4:** Input variables used for the KAIST dataset.

Input Variable	Signal Description	Sampling Frequency
$X_{1}$	Bearing housing A, *x*-direction vibration	25.6 kHz
$X_{2}$	Bearing housing A, *y*-direction vibration	25.6 kHz
$X_{3}$	Bearing housing B, *x*-direction vibration	25.6 kHz
$X_{4}$	Bearing housing B, *y*-direction vibration	25.6 kHz

**Table 5:** Default parameter settings of PHDGCN.

Parameter	Symbol	CWRU	KAIST
Window length	*L*	16	64
Window stride	*s*	4	16
FFT dimension	*F*	9	33
Historical window number	*K*	7	7
Temporal decay coefficient	τ	2.0	2.0
Graph hidden dimension	*H*	64	64
Topology residual hidden dimension	$H_{e}$	128	128
Fusion hidden dimension	$H_{f}$	64	64
Temporal refinement coefficient	$\lambda_{\mathrm{tem}}$	0.5	0.5
Covariate refinement coefficient	$\lambda_{\mathrm{cov}}$	0.5	0.5
Covariate residual floor	$\epsilon_{c}$	0.05	0.05
Topology regularization coefficient	β	$10^{-3}$	$10^{-3}$
Dropout rate	–	0.2	0.2
Batch size	–	64	256
Learning rate	–	$10^{-3}$	$10^{-3}$
Weight decay	–	$10^{-5}$	$10^{-5}$
Maximum epochs	–	200	200
Early-stopping patience	–	35	35

Note: Due to the different sampling frequencies of the CWRU and KAIST datasets, the window-related parameters are adjusted accordingly, while the core model settings remain unchanged.

**Table 6:** Key parameter settings of the comparison methods.

Method	Key Parameter Settings
MLP	Two hidden layers; hidden dimension H=64; dropout =0.2.
LSTM	Two LSTM layers; hidden dimension H=64; dropout =0.2.
CNN	Two 1-D convolutional layers; hidden channels =64; kernel size =3; dropout =0.2.
GCN	Two graph convolutional layers; hidden dimension H=64; training-set Pearson correlation graph; dropout =0.2.
TCN	Two residual temporal blocks; hidden channels =64; kernel size =3; dilation rates {1,2}; dropout =0.2.
SENGraph	Hidden dimension H=64; fine temporal steps =4; graph evolution layers =2; dropout =0.2.
ASG-GTCN	Hidden dimension H=64; GraphSAGE layers =3; attention heads =4; spline knots {0.2,0.4,0.6,0.8}; GTCN dilation rates {1,2}; dropout =0.2.

**Table 7:** Comparison results and inference time on the CWRU and KAIST datasets.

Method	CWRU			KAIST		
	ACC (%)	F1 (%)	Time (ms)	ACC (%)	F1 (%)	Time (ms)
MLP	90.64	89.97	**0.14**	84.66	84.34	**0.12**
LSTM	86.66	85.89	0.26	80.06	77.40	0.23
CNN	90.11	89.70	0.29	82.21	80.48	0.22
GCN	82.68	82.03	0.32	82.21	81.83	0.28
TCN	89.94	89.56	0.56	84.36	81.54	0.44
SENGraph	86.48	85.74	2.52	80.67	78.23	2.14
ASG-GTCN	89.23	88.66	3.92	84.36	82.41	3.50
PHDGCN	**93.75**	**93.34**	3.12	**89.47**	**87.08**	2.94

Note: Bold values indicate the best results for each metric. For inference time, the lowest value is considered the best.

**Table 8:** Ablation results on the CWRU and KAIST datasets.

Variant	CWRU		KAIST	
	ACC (%)	F1 (%)	ACC (%)	F1 (%)
A1: w/o Temporal Dynamic	88.82	88.20	80.98	78.37
A2: w/o Covariate Dynamic	88.36	87.18	80.67	80.02
A3: Fixed Dual-View	88.30	87.31	81.29	78.66
A4: Temporal Only	89.29	88.52	85.28	83.40
A5: Covariate Only	72.56	71.20	84.97	83.81
A0: Full	**93.75**	**93.34**	**89.47**	**87.08**

Note: Bold values indicate the best results for each metric.

## Data Availability

The CWRU and KAIST datasets used in this study are publicly available from their respective original repositories.

## Abbreviations

The following abbreviations are used in this manuscript:

PHDGCN	Prior-Guided Heterogeneous Dynamic Graph Convolutional Network
GCN	Graph Convolutional Network
GNN	Graph Neural Network
FFT	Fast Fourier Transform
MLP	Multilayer Perceptron
LSTM	Long Short-Term Memory
CNN	Convolutional Neural Network
TCN	Temporal Convolutional Network
BPFI	Ball-Pass Frequency of the Inner Race
BPFO	Ball-Pass Frequency of the Outer Race

## References

[1] Yang Z., Ge Z. (2022). On Paradigm of Industrial Big Data Analytics: From Evolution to Revolution. IEEE Trans. Ind. Inform..

[2] Xu Y., He D., Luo X., Jin Z., Sun H. (2026). CSSCL: A cross-sensor self-contrastive learning for nondestructive fault detection of heavy locomotive motors in label-scarce scenarios. Nondestruct. Test. Eval..

[3] Hu X., Jiang C., Huang Y., Peng D., Su H., He Y., Chen Z. (2026). SMNet: A novel compositional generalization model for industrial robot multi-joint fault diagnosis. IEEE Internet Things J..

[4] Wang H., Liu X. (2024). Research on Rotating Machinery Fault Diagnosis Based on Improved Multi-Target Domain Adversarial Network. Instrumentation.

[5] Yang Z., Chen J., Bi Z., Jiang X., Yao L., Lou J., Song Z. (2026). CLARI: Constrained Reflective LLM Inference for Few-Shot Soft Sensing under Operating-Condition Drift. Control Eng. Pract..

[6] Rehman A.U., Jiao W., Jiang Y., Wei J., Sohaib M., Sun J., E S., Rehman K.u., Chi Y. (2025). Deep Learning in Industrial Machinery: A Critical Review of Bearing Fault Classification Methods. Appl. Soft Comput..

[7] Hu X., Zhang H., Cao J., Huang Y., Zhang X., Wang H., Tang T. (2026). PSRONet: A deep reinforcement learning-based sensor configuration framework in railway point machines fault diagnosis. IEEE Trans. Instrum. Meas..

[8] Jiang X., Xie H., Wang J., Yang Z., Zhou Y., Yao L., Zhu Z. (2026). Agentic AI for Safety-Aware Process Monitoring and Fault Diagnosis: A Review. Processes.

[9] Ding Y., Cao Y., Jia M., Ding P., Zhao X., Lee C.G. (2024). Deep Temporal–Spectral Domain Adaptation for Bearing Fault Diagnosis. Knowl.-Based Syst..

[10] Zou X., Zhang H., Jiang Z., Zhang K., Xu Y. (2024). Toward Accurate Extraction of Bearing Fault Modulation Characteristics with Novel Time–Frequency Modulation Bispectrum and Modulation Gini Index Analysis. Mech. Syst. Signal Process..

[11] Shi L., Guo J., Wang H. (2024). Precision Machining Equipment Fault Diagnosis Based on CWT and Improved ResNeXt. Instrumentation.

[12] Yang C., Yan J., Feng Y., Li L., Tan J. (2026). Hybrid Deep Learning for Hydraulic Cylinder Fault Diagnosis under Complex Conditions via Multi-Source Signal Fusion. Instrumentation.

[13] Wu Z., Pan S., Chen F., Long G., Zhang C., Yu P.S. (2021). A Comprehensive Survey on Graph Neural Networks. IEEE Trans. Neural Netw. Learn. Syst..

[14] Tang M., Yao L., Zhu Z., Shen B., Zeng J., Song Z. (2026). Semi-supervised spatio-temporal graph variational autoencoder for enhanced industrial process fault diagnosis under label scarcity. Control Eng. Pract..

[15] Jia M., Yao Y., Liu Y. (2025). Review on Graph Neural Networks for Process Soft Sensor Development, Fault Diagnosis, and Process Monitoring. Ind. Eng. Chem. Res..

[16] Liu Y., Shen B., Wong D.S.H., Jia M., Yao Y. (2026). Explainable Neural Network Meets Graph Neural Network: Recent Advances in Process Fault Detection and Diagnosis. Comput. Chem. Eng..

[17] Li Y., Liu X., Hu J., Liang P., Wang B., Yuan X., Zhang L. (2025). Graph Optimization Algorithm Enhanced by Dual-Scale Spectral Features with Contrastive Learning for Robust Bearing Fault Diagnosis. Knowl.-Based Syst..

[18] Dai C., He D., Jin Z., Zhang X., Chen G., Wu J., Zhao J., Xu Y. (2026). Digital twin-assisted graph contrastive domain adaptation for small-sample bearing fault diagnosis. Struct. Health Monit..

[19] Cai W., Zhao D., Wang T. (2025). Spatial-Temporal Graph Attention Contrastive Learning for Semi-Supervised Bearing Fault Diagnosis with Limited Labeled Samples. Comput. Ind. Eng..

[20] Wang J., Xiong W., Yao L., Jiang X., Cui X., Yu W., Young B. (2026). GraphSiamese: Novel Spatial-Temporal Slow Feature Fusion Network for Industrial Process Quality Prediction. Appl. Soft Comput..

[21] Huang Y., Cui B., Mao X., Yang J. (2024). Prior Knowledge-Informed Graph Neural Network with Multi-Source Data-Weighted Fusion for Intelligent Bogie Fault Diagnosis. Machines.

[22] Xiao Y., Liu D., Cui L., Wang H. (2024). Heterogeneous Graph Representation-Driven Multiplex Aggregation Graph Neural Network for Remaining Useful Life Prediction of Bearings. Mech. Syst. Signal Process..

[23] Mao L., Yang Z., Shen B., Jiang X., Yao L., Song Z. (2026). Edge-Compressed and Energy-Optimized Dual-Domain Graph Network with Variational Gating for Industrial Soft Sensing. IEEE Sens. J..

[24] Cai W., Zhao D., Wang T. (2025). Multi-Scale Dynamic Graph Mutual Information Network for Planet Bearing Health Monitoring under Imbalanced Data. Adv. Eng. Inform..

[25] Yang Z., Mao L., Ye L., Ma Y., Song Z., Chen Z. (2025). AKGNN: When Adaptive Graph Neural Network Meets Kolmogorov–Arnold Network for Industrial Soft Sensors. IEEE Trans. Instrum. Meas..

[26] Jin Z., Wu Z., He D., Zhao J., Fu Y. (2026). Data-driven nondestructive evaluation for bearing prognostics using a dual correlation graph knowledge distillation network. Nondestruct. Test. Eval..

[27] Hu X., Zhang K., Huang Y., Cao J., Peng D., Chen Z., Tang T. (2026). RRDNet: Robust fault diagnosis of railway switch machines under imperfect sensing. IEEE Trans. Instrum. Meas..

[28] Yan F., Wang C., Wang Z., Shen Y., Yang C. (2025). SENGraph: A Self-Learning Evolutionary and Node-Aware Graph Network for Soft Sensing in Industrial Processes. IEEE Trans. Neural Netw. Learn. Syst..

[29] Hui Y., Han Y., Zhao X. (2026). ASG-GTCN: Spatiotemporal Soft Sensor Based on Adaptive Spline-Driven Dynamic Graph Learning for Industrial Processes. J. Taiwan Inst. Chem. Eng..

[30] Zheng C., Zong B., Cheng W., Song D., Ni J., Yu W., Chen H., Wang W. (2020). Robust Graph Representation Learning via Neural Sparsification. Proceedings of the 37th International Conference on Machine Learning, Virtual, 13–18 July 2020.

[31] Karniadakis G.E., Kevrekidis I.G., Lu L., Perdikaris P., Wang S., Yang L. (2021). Physics-Informed Machine Learning. Nat. Rev. Phys..

[32] Wang D., Cao C., Chen N., Pan W., Li H., Wang X. (2023). A correlation-graph-CNN method for fault diagnosis of wind turbine based on state tracking and data driving model. Sustain. Energy Technol. Assess..

[33] Smith W.A., Randall R.B. (2015). Rolling Element Bearing Diagnostics Using the Case Western Reserve University Data: A Benchmark Study. Mech. Syst. Signal Process..

[34] Jung W., Kim S.H., Yun S.H., Bae J., Park Y.H. (2023). Vibration, Acoustic, Temperature, and Motor Current Dataset of Rotating Machine under Varying Operating Conditions for Fault Diagnosis. Data Brief..

[35] Kipf T.N., Welling M. Semi-Supervised Classification with Graph Convolutional Networks. Proceedings of the International Conference on Learning Representations.

[36] Bai S., Kolter J.Z., Koltun V. (2018). An Empirical Evaluation of Generic Convolutional and Recurrent Networks for Sequence Modeling. arXiv.

[37] Selvaraju R.R., Cogswell M., Das A., Vedantam R., Parikh D., Batra D. Grad-cam: Visual explanations from deep networks via gradient-based localization. Proceedings of the IEEE International Conference on Computer Vision.

